# Genome-Wide Analysis of TIR-NBS-LRR Gene Family in Potato Identified *StTNLC7G2* Inducing Reactive Oxygen Species in Presence of *Alternaria solani*

**DOI:** 10.3389/fgene.2021.791055

**Published:** 2022-01-10

**Authors:** Namo Dubey, Anjali Chaudhary, Kunal Singh

**Affiliations:** ^1^ CSIR-Institute of Himalayan Bioresource Technology, Palampur, India; ^2^ Academy of Scientific and Innovative Research (AcSIR), Ghaziabad, India

**Keywords:** potato, *Alternaria solani*, TIR-NBS-LRR, early blight disease, plant defense

## Abstract

Resistance gene analogs (RGAs) comprising NBS-LRR gene family members are considered prominent candidates in the development of disease-resistant genotypes. NBS-LRR gene family comprised a very large number of genes; therefore, members of one subfamily TIR-NBS-LRR (TNL) are identified in the present study from *Solanum tuberosum* genome, followed by their bioinformatics characterization. The study identified a total of 44 genes encoding 60 TNL transcripts with two prominent clusters at chromosome 1 and chromosome 11. Expression analysis of 14 TNL genes after *Alternaria solani* infection at 1, 2, 3, 5, and 7 days post inoculation in two disease-tolerant varieties, Kufri Jyoti and Kufri Pukhraj, and one relatively susceptible variety, Kufri Chandramukhi, showed differential expression of many genes including a high expression (>15-fold) of *StTNLC6G2T1* and *StTNLC11G9T1*. Functional characterization of one such gene, *StTNLC7G2*, reveals involvement in the generation of reactive oxygen species under *A. solani* attack, implicating its putative role in plant defense *via* hypersensitive response.

## Introduction

Phytopathogens are microbes that have developed the capacity to suppress and overcome the host immune responses and inhabit the plant tissue for their survival, growth, and even multiplication (Chisholm et al., 2006). In response, plants have evolved too with time and developed a sophisticated immune system involving specialized resistance proteins to cope and combat a wide array of pathogens (Qi and Roger, 2013). Members of the NBS-LRR family are one such class of proteins consisting of an N-terminal CC or TIR domain, central NBS domain, and LRR motifs at C-terminal (Mchale et al., 2006). These proteins, also known as resistance gene analogs (RGAs), have been studied extensively in many plant systems with delineation of their role against a variety of pathogens including fungal, viral, nematode, and bacterial species (Dubey and Singh, 2018). In plants, they form a major line of defense under innate immunity. Plants' innate immunity is the first line of defense response to pathogen attack by recognition of pathogen-associated molecular patterns (PAMPs) by plant recognition receptors (PRRs) (Jones and Dangl, 2006). This is followed by the induction of reactive oxygen species (ROS) known as hypersensitive response (HR) and recognition of pathogen-associated virulence factors and effectors by members of the NBS-LRR protein family. The recognition and sensing of pathogen attack along with downstream relay of a signal by defense-associated signaling pathway help the host to act decisively (Padmanabhan et al., 2009). One major subset of this protein family composed of members with toll-interleukin receptor (TIR) domain at the N-terminal is known as TIR-NBS-LRR (TNL) proteins (Nandety et al., 2013).

Many TNLs have been reported to provide resistance in plants against microbial pathogens (Whitham et al., 1994). One such protein identified is Y1 of potatoes against potato virus Y (PVY), a homolog of N protein of tobacco that confers cell death upon infection with PVY (Goyer et al., 2015). Therefore, TNL proteins are prime candidates in assisting plant defense against phytopathogens. Among the various pathogens infecting potatoes (*Solanum tuberosum* L.), the filamentous pathogens including oomycetes and fungi are considered the most devastating. Early blight disease caused by *Alternaria solani*, late blight of potatoes caused by *Phytophthora infestans*, and *Rhizoctonia solani* causing black scurf and stem canker of potatoes are prominent ones. *A. solani* can be highly devastating in regions with frequent rainfall with warm and dry conditions, leading to huge losses to farmers (Vloutoglou and Kalogerakis, 2000).

In the present study, TNL genes encoding putative proteins have been identified from the potato phureja genome and characterized by bioinformatics methodology. Selected transcripts were analyzed for expression behavior against fungal phytopathogen *A. solani*. One gene, *StTNLC7G2*, showing induced expression after pathogen attack and localization in the plasma membrane was further characterized *via* agroinfiltration in *S. tuberosum* as well as *Nicotiana benthamiana*.

## Materials and Methods

### Plant Material and *Alternaria solani* Infection

Three potato genotypes, namely, Kufri Pukhraj (KP), Kufri Jyoti (KJ), and Kufri Chandramukhi (KC), were procured. All the three varieties were grown under an institutional farm field at CSIR-IHBT (latitude 32.0934°N; longitude 76.5439°E), Palampur, India. Among the three varieties used in the experiments, KJ and KP have been previously released as blight disease-tolerant varieties, while KC is considered more susceptible to blight diseases of potatoes (https://cpri.icar.gov.in/AddLink/getIMG/6068). The *A. solani* (MTCC-10690; accession no. HM484353.1) culture was procured from IMTECH, Chandigarh, India, maintained in a laboratory at 25°C, and used for infection purposes. The fungal culture was inoculated to 1-month-old susceptible potato plants with 100 ml of fungal suspension (2 × 10^5^ conidia ml^−1^) and sprayed to retain the virulence. Conidia were counted using hemocytometer under a microscope. Plants were kept at a relative humidity of 70% at 26°C with 12-h day/night condition. Fungal mycelia obtained from infected portion using single spore culture methodology (Choi et al., 1999) were used for further maintenance of culture along with subsequent infection work. The identity of fungus being *A. solani* was further confirmed with the use of Internal Transcribed Spacer (ITS) sequencing using universal primer pairs ITS1–ITS4. The 556-bp ITS sequence is shown in Supplementary Figure S2A. *A. solani* biomass was measured for infected plants of each variety using fungal-specific primer pairs (AS1 and AS2) by qRT-PCR. The methodology of biomass measurement was followed as described by Kumar et al. (2013).

### Identification of Putative TIR-NBS-LRR Proteins

Total proteome (39,031) was downloaded from the PGSC database and used for the screening of TIR-NBS domain family (PGSC_DM_v3.4_pep_representative.fasta.zip://potatogenomics.plantbiology.msu.edu/index.html). A set of putative TIR-NBS proteins was identified from the complete potato proteome using hidden Markov model (HMM) profiles as previously described (Arya et al., 2014). To improve the numbers of TNL proteins left out by HMM application, another strategy was applied that uses pfam ids and conserved domain (CD) information using the CD database (CDD) of the National Center for Biotechnology Information (NCBI) (Supplementary Figure S1). All the proteins obtained were also cross-checked using MyHits (Pagni et al., 2004) and ScanProsite (de Castro et al., 2006) along with manual curation to ensure the presence of all the three TNL domains. Each transcript was further assessed for LRR motifs manually, using LxxLxLxxN/CxL or LxxLxL consensus (Enkhbayar et al., 2004), where x denoted any amino acid and L signifies leucine, along with LRR pfam id-0562. To rule out the presence of CC domain, MARCOIL HMM with a threshold of 90 was used (Delorenzi and Speed, 2002). “PGSC_DM_v3.4_g2t2c2p2func.txt” file was used to retrieve information regarding TNLs encoding desired transcripts. To examine the structural motifs among the identified TNLs, the predicted protein sequences were submitted for the motif analysis by MEME version 4.9.0 (Bailey et al., 2009).

### Physical Mapping and Gene Duplication Events

The scaffold for all twelve chromosomes was obtained from the PGSC database and used for mapping TNL genes. The General Feature Format (GFF) file (PGSC_DM_v3.4_gene.gff) was also retrieved. These TNL genes were graphically portrayed on the chromosome using “PhenoGram” tool of Ritchie Lab (http://visualization.ritchielab.psu.edu/phenograms/plot). To check possible duplicated TNLs in potatoes, the MCScanX program (Wang et al., 2012) was applied with all-vs-all blastp using parameters *B* = 100, *V* = 10, *E*-value 1e−10, and tabular output format.

### Phylogenetic Analysis of TIR-NBS-LRRs

A phylogenetic tree was prepared using the longest peptide of each TNL gene. The TNL protein sequences were aligned using Clustal W incorporated in MEGA v.7.0 (http://www.megasoftware.net), and a phylogenetic tree was prepared using the neighbor-joining (NJ) method with a bootstrap of 1,000 iterations (Zhang et al., 2018).

### Quantitative Real-Time PCR

About 100 ml of fungal suspension (2 × 10^5^ conidia ml^−1^) was sprayed on plants. The concentration of conidia and mycelium pieces was calculated using a hemocytometer (Sigma-Aldrich Corp., St. Louis, MO, USA) under a microscope. Sterile water (Mock)-treated plants were used as control. All the plants were kept in a growth chamber with a relative humidity of 70% and temperature of 26°C with 12-h day/night conditions. Leaf samples from each potato cultivars were collected at five time points, *viz*., 1, 2, 3, 5, and 7 days post inoculation (dpi), from three biological replicates of pathogen-inoculated (treatment) and mock-inoculated (control) plants. Total RNA was isolated as previously described (Ghawana et al., 2011) from leaf tissues collected at different time points. The RNA was reverse transcribed using High Capacity cDNA synthesis kit (Applied Biosystems, Foster City, CA, USA). The cDNA was diluted 10-fold with diethyl pyrocarbonate (DEPC)-treated water. Primer Express software version 3.0.1 (Thermo Fisher Scientific, Waltham, MA, USA) was used to design the primers. The primers were designed with an amplicon size range of 80–190 bp, primer length of 18–25 nucleotides, Tm of 50–60°C, and maximum guanine–cytosine (GC) content of 50%–60%. All primer sequences are presented in Supplementary Table S1. The Power Sybr Green PCR master mix (ABI) was utilized for the gene expression using an optical 96-well plate on MX3000P real-time PCR machine (Agilent Technologies, Waldbronn, Germany) as per the manufacturer’s manual. Elongation factor (*StEf1a*) (accession no. PGSC0003DMG400023270, XM_006343393.2) was selected as reference gene to normalize the targeted gene expression (Tang et al., 2017). The relative expression was calculated by the 2^−∆∆CT^ method (Schmittgen and Livak, 2008). All the experiments were repeated thrice with the inclusion of no template control (NTC) for the detection of DNA contamination.

### Localization Assessment of *StTNLC7G2* (*StTNL41857*)

The full-length gene was amplified and cloned in-frame with C-terminal of green fluorescent protein (GFP) in vector pCAMBIA1302 and recombinant vector carrying GFP: *StTNLC7G2* construct was introduced into *Agrobacterium* GV3101 and incubated with onion peel epidermis (Sun et al., 2007). After 2 days, the epidermal layer was thoroughly washed with distilled water and visualized under a fluorescence microscope (Evos, Thermo Fisher Scientific) at 40×.

### Functional Characterization of *StTNLC7G2 via* Agroinfiltration

Conidial suspension of *A. solani* was prepared using 0.01% Tween-80, and 50 μl was pre-inoculated (painted) on the proposed infiltration site and dried afterward for 30 min. The gene was cloned in pCAMBIA1302 using *Nco*I and *Bst*EII (Supplementary Figure S2B) and confirmed by sequencing (accession no. MZ597472). The positive *Agrobacterium* transformants were used for culturing in 200 ml of yeast extract peptone broth (YEP) media supplemented with rifampicin (25 μg/ml) and kanamycin (50 μg/ml) at 28°C to an OD_600_ of ~1.0. The culture was further centrifuged at 4,000×*g* for 10 min, and the pellet was resuspended in 10 mM of MES [2-(*N*-morpholino)ethanesulfonic acid] pH 5.6 and 10 mM of MgCl_2_ with 100 µM of acetosyringone (Bhaskar et al., 2008) and incubated for 4–8 h at room temperature (RT) in the dark, and 200 µl of the inoculum was used for infiltration of 30-day-old potato leaves of KJ, KC, and KP. The *Agrobacterium* transformed with a blank vector was used as vector control. The inoculated plants were kept in the dark for 12 h, then shifted in a plant growth chamber with conditions described earlier, and sampled at 3 dpi. For DAB staining, 50 mg/100 ml of working stock was prepared in 0.1 M of sodium phosphate buffer at pH 3.0 and was maintained by using concentrated HCl. Earlier collected samples were now dipped into the staining solution and incubated at RT for 6–8 h at 100 rpm. Then leaves were decolorized using the bleaching solutions (ethanol:acetic acid:glycerol, 3:1:1) for 30 min at 90°C, then replaced with fresh bleaching solutions, and photographed for any brown color appearance denoting ROS (Liu et al., 2014). Gene expression after agroinfiltration was confirmed in *N. benthamiana* leaves collected at 5 dpi. Samples were collected by an incision measuring 1 × 1 cm^2^ using a razor blade surrounding the infiltration zones (2–3 cm away from the infiltration zones). RT-PCR was performed as previously described (Bhaskar et al., 2008).

## Results

### TIR-NBS-LRR Genes Were Identified Encoding 60 Full-Length Peptides

The application of Hmmsearch method using Pf00931-pfam domain (NBS) resulted in 644 NBS coding transcripts. The application of TIR Pfam id-1582 within these transcripts narrowed down the result to 110 TIR-NBS-containing transcripts. After assessment of LRR motifs, in total, 55 transcripts were identified containing all the three domains, namely, TIR, NBS, and LRR. As there is always a possibility to skip the detection of the targeted domain by HMM profile due to partial or truncated sequences, a second strategy of CD search using the entire proteome set from the PGSC database was submitted on the NCBI CDD search engine. The strategy provided a total of 60 full-length TNL peptides with a high confidence level for TIR, NBS, and LRR domain encoded by 44 TNL genes (Supplementary Table S2). When these 44 genes were used to identify all possible coded peptides, a total of 36 additional transcripts were identified including 29 truncated sequences. Twenty-seven genes were found to code for more than 1 transcript probably by alternative splicing events (Supplementary Table S3). In MEME analysis, conserved and signature motifs of the TIR domain and NBS domain were identified to be preserved (Supplementary Table S4).

### Novel Methodology for Gene Family Member Nomenclature

Nomenclature has been assigned to these TNL genes for ease of understanding with an effort to bring uniformity of methodology in such study after retrieving relevant information in a smooth and easy manner. Here, the nomenclature of individual genes and their respective proteins is based on their physical location on the chromosome. For instance, *PGSC0003DMG400022699* localized on chromosome 1, at the first position, so the nomenclature of this gene and encoded protein is *StTNLC1G1* and StTNLC1G1P1, respectively. If a gene encodes more than one transcript, the nomenclature used here is based on their peptide length in descending order. For example, *PGSC0003DMG400024055* encodes 2 peptides: PGSC0003DMP400041617 and PGSC0003DMP400041616. The nomenclature of these peptides will be StTNLC1G2P1 and StTNLC1G2P2, respectively (Supplementary Table S2).

### Phylogenetic Analyses

**FIGURE 1:**
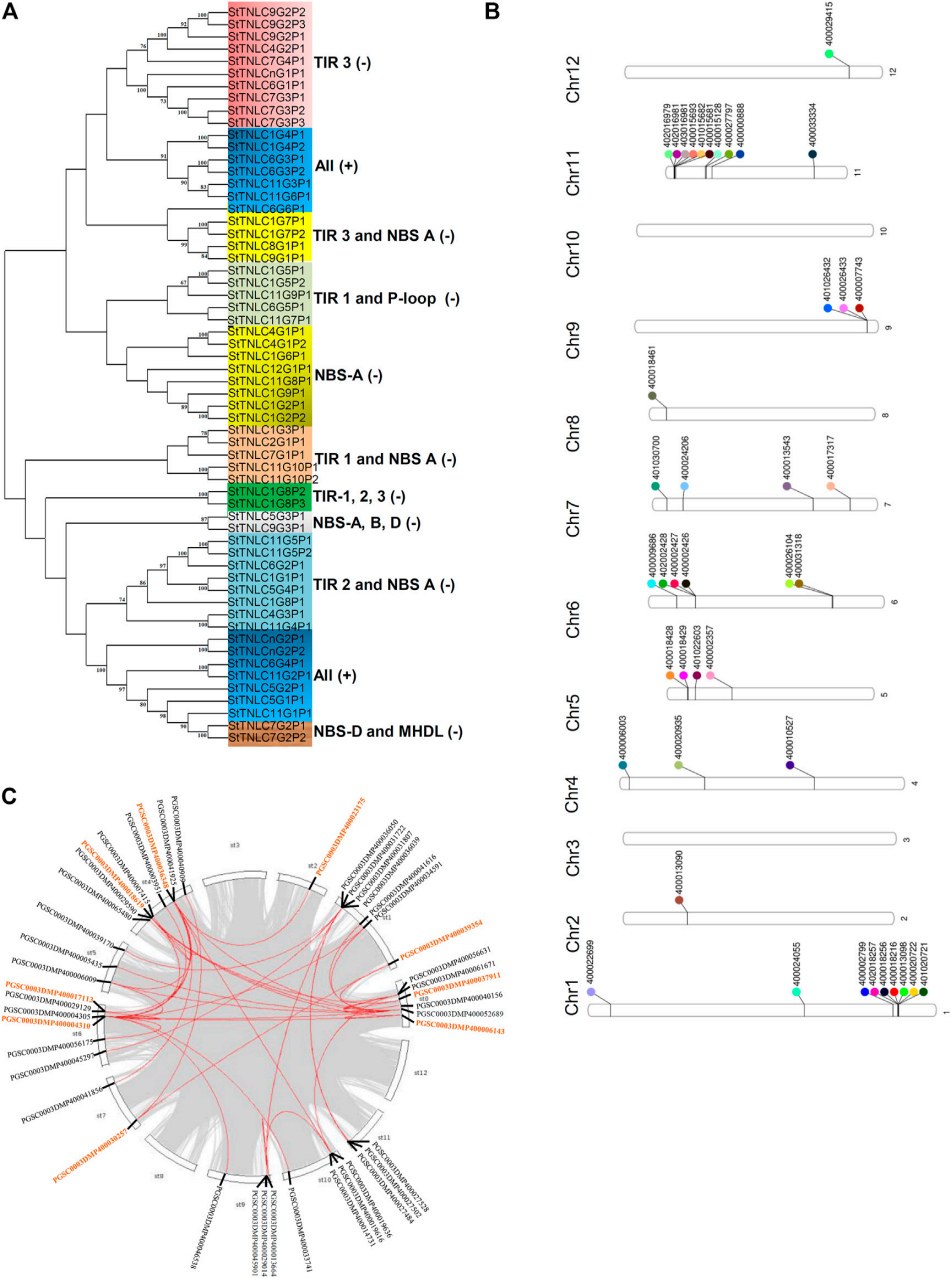
**(A)** Phylogenetic tree of 60 TIR-NBS-LRR (TNL) peptides showing clades getting grouped and demarcated based on the presence of TIR and NBS motifs. **(B)** Phenogram representing the position of 44 TNLs on chromosomes of *Solanum tuberosum*. **(C)** *StTNL* collinear gene pairs within a genome are represented with red lines, while the genes involved in *StTNL*–*StTNL* duplication events are represented with orange color in the text.

Phylogenetic grouping of the genes from the same chromosomes and the separate chromosomes was observed. Exon–intron structure tends to remain the same among genes in the same clade, indicating significant gene structure conservation over evolution, according to a phylogenetic tree–gene structure correlation. Clades were also formed on the basis of the presence and the absence of a particular motif in proteins and homology between them. Two major clades were formed, i.e., Clade I and Clade II, in which TNLs were demarcated on the basis of the domain and motif compositions (Figure 1A).

### Chromosomal Localization of *StTNL* Genes

The physical location of the TNL genes was resolved on the basis of PGSC_DM_v3.4_gene.gff file as retrieved from the PGSC database. Forty-two out of forty-four TNL genes were successfully mapped on the twelve potato chromosomes using Ritchie Lab phenogram, while only 2 sequences remained unanchored. The chromosomal location of these genes has shown the uneven distribution of TNLs on different chromosomes. Two gene clusters were found, one each on chromosomes 1 and 11 (Figure 1B), both having 6 TNL genes. The number of TNLs mapped on chromosomes 1, 2, 4, 5, 6, 7, 8, 9, 11, and 12 was 9, 1, 3, 4, 6, 4, 1, 3, 10, and 1, respectively. There were no TNL genes found on chromosomes 3 and 10.

### Duplication Events Observed

Across the potato genome, there were 7 pairs of TNL-TNL segmental duplication events contributed by a total of 9 TNL genes. *StTNL36348* was found to be involved in three TNL-TNL events with pairing to *StTNL17113*, *StTNL18619*, and *StTNL23175* in the genome. Two genes, *StTNL37911* and *StTNL4310*, are probably segmentally duplicated at least six times, including non-TNL duplications. The search for duplication events of TNL vs. other genes identified 35 duplication events/pair. Collinear pair was found on all chromosomes except chromosomes 3, 8, and 12 (Figure 1C). Only one gene, *StTNL45901*, physically located on chr9 was observed to be tandemly duplicated (Supplementary Table S5).

### Expression of Multiple *StTNL* Genes Induced by Fungal Pathogen *Alternaria solani*

**FIGURE 2:**
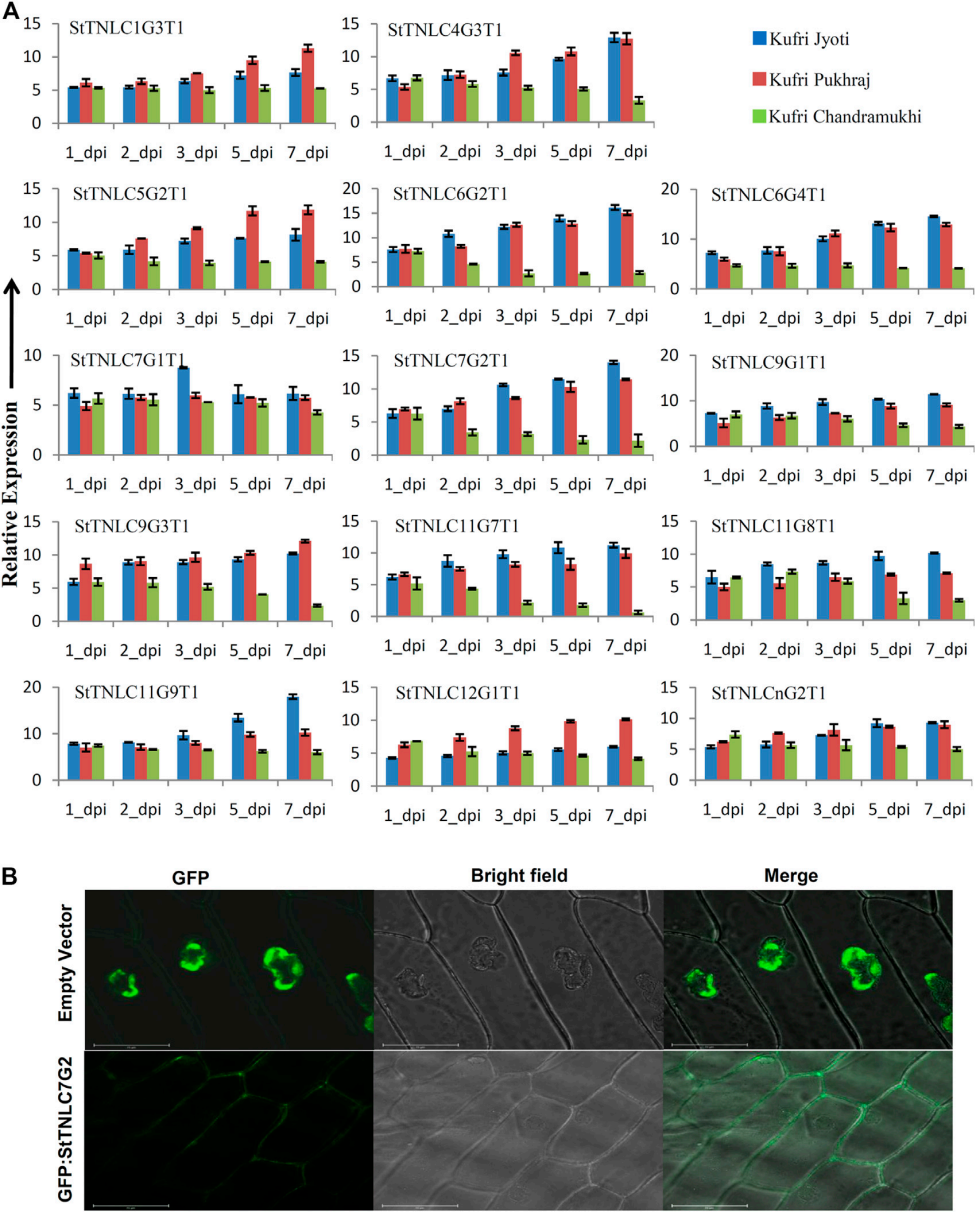
**(A)** Differential expression of 14 TIR-NBS-LRRs (TNLs) following *Alternaria solani* infection. The expression values were normalized with *StELF1α* as an internal control. Relative expression values of each transcript were assessed in three varieties at five time points. The error bar shows the standard error (SE) with *n* = 3. **(B)** Fluorescence microscopy observation of green fluorescent protein (GFP): *StTNLC7G2* construct after expression in onion leaf epidermal cell. Vector with GFP only was taken as a control for localization comparison and assessment.

Before proceeding for expression assessment of transcripts, the virulence of *A. solani* was confirmed by visualization of symptoms using pathogen spray to plants in pots (Supplementary Figure S4A) and detached leaf assay (Supplementary Figure S4B) at 5 dpi. Both the experiments confirmed the susceptibility of KC to virulent *A. solani*, while KJ and KP showed tolerance. The results were even further supplemented with fungal biomass assessment (Supplementary Figure S4C). Fourteen genes were chosen randomly for expression analysis, but also keeping in mind that at least one gene from each chromosome may be represented. The differential expression pattern obtained for different transcripts can be summed up in three major patterns for TNL transcripts. The first groups of transcripts have their expression level constantly reaching more than 4.0-fold in expression across all the three varieties (Supplementary Tables S6–S8). Such expression pattern was obtained for seven transcripts, namely, *StTNLC1G3T1*, *StTNLC6G4T1*, *StTNLC7G1T1*, *StTNLC9G1T1*, *StTNLC11G9T1*, *StTNLC12G1T1*, and *StTNLCnG2T1*. All of them showed more than 10-fold expression in at least one disease-tolerant variety with the exception being *StTNLCnG2T1* and *StTNLC7G1T1* (Figure 2A). Strong and constant upregulation under pathogen treatment suggests that their expression modulates under pathogenic signals like pathogen-associated molecular pattern (PAMP). A few transcripts showed a difference in expression pattern between tolerant and susceptible varieties. Transcripts with such expression behavior show a constant decrease in expression in KC after the initial spike at 1 or 3 dpi. These transcripts were *StTNLC6G2T1*, *StTNLC7G2T1*, *StTNLC9G3T1*, *StTNLC11G7T1*, and *StTNLC11G8T1*. The expression of all these transcripts was decreased to <2.5-fold in KC at 7 dpi while maintaining >6.0-fold expression in KP and KJ.

Meanwhile, two transcripts showed expression of >15.00-fold under *A. solani* infection. These transcripts were *StTNLC6G2T1* and *StTNLC11G9T1*. The difference in expression value of these two genes at 7 dpi was almost 4 times in the comparison of KJ with KC. *StTNLC6G2T1* and *StTNLC11G9T1* must have prominent roles in plant defense under *A. solani* attack at a late stage. For the majority of the genes tested, the expression enhancement pattern was almost similar for KJ and KP with only a significant difference being obtained in *StTNLC12G1T1*. Furthermore, among all the candidates tested, the expression pattern of only one transcript (*StTNLC7G1T1*) was found to be constant across the varieties and time points.

### *StTNLC7G2* Induced More in Tolerant Variety and Localizes to Plasma Membrane

One gene, *StTNLC7G2*, showing homology with *N* gene of tobacco was selected for further characterization, as its transcript induction was greater than 10-fold at 5 and 7 dpi in KJ and KP, respectively, while expression in KC barely crosses 2-fold with respect to control, though immediately after pathogen inoculation at 1 dpi, the expression was similar in all three varieties. To check the localization of StTNLC7G2, the transcript was amplified and cloned in pCAMBIA1302 in the same frame as of GFP, and positive clones were used to inoculate onion peel. Amplification and cloning of full-length *StTNLC7G2* achieved 1,296 bp of amplicon encoding a protein of 431 amino acids (Supplementary Figure S2C). The localization of construct was compared with vector control (GFP only) that clearly showed nucleus localization, while StTNLC7G2 protein showed putative membrane localization (Figure 2B). StTNLC7G2 protein is probably involved in the reception of the pathogenic signal and their further transmission downstream. The high expression of the transcript at 7 dpi in a tolerant variety further indicates the same.

### StTNLC7G2 Induces Reactive Oxygen Species Under *Alternaria solani* Attack

**FIGURE 3:**
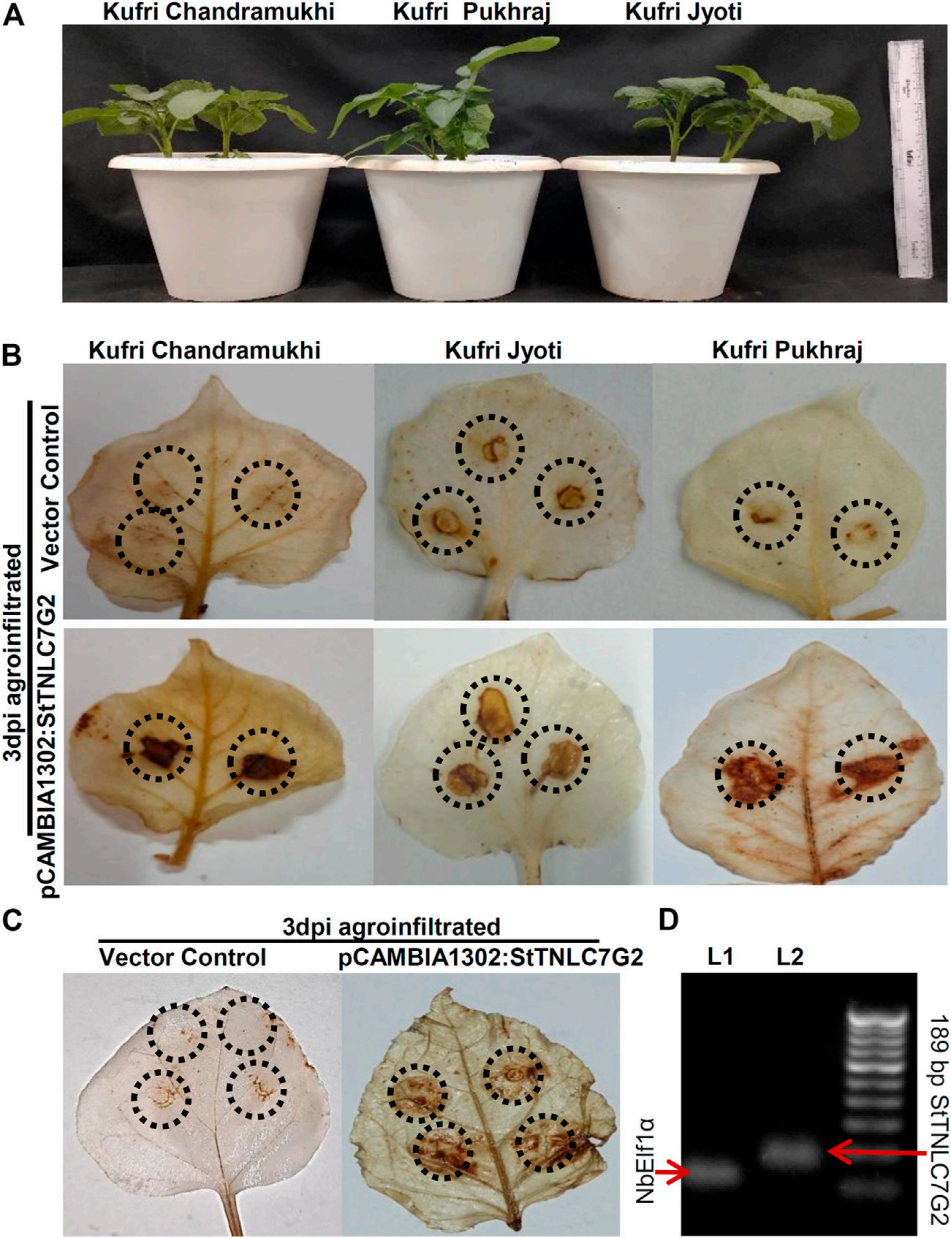
Agroinfiltration assays of *StTNLC7G2* gene in potato plants. **(A)** One-month-old potato plants selected for each variety for agroinfiltration with nearly the same size. **(B)** Leaf images showing dark patches and spots at 3 dpi after DAB staining indicating induction of reactive oxygen species in response to *StTNLC7G2* under *Alternaria solani* attack in three varieties of potato. **(C)** Confirmation of reactive oxygen species generation under *StTNLC7G2* influence in *Nicotiana benthamiana* in comparison with vector control. **(D)** Gel image showing high expression of *StTNLC7G2* transcripts L2 (lane 2: 189 bp) from *Nicotiana* leaves 5-days post agroinfiltration. In L1 (lane 1: 117 bp), *NbElf1α* was taken as internal control.

After inoculation of the fungal pathogen, *A. solani* suspension in the leaves, agroinfiltration was performed with both positive gene construct and vector control at the same site and left for visual observation followed by sampling. DAB staining of 3 dpi agroinfiltrated leaves showed dark patches in all the three varieties at 3 dpi (Figure 3A), indicating the prominent generation of ROS in the presence of StTNLC7G2 mimicking HR (Figure 3B; Supplementary Figure S4D). KJ showed light-dark patches in mock-treated (empty vector) leaves too, suggesting a stronger defense mechanism available to its genotype. An increase in ROS induction in the presence of StTNLC7G2 was more prominent in KP and KC. As ROS induction is the primary feature of HR during plant defense, the *StTNLC7G2* is probably co-related with innate immunity against fungal pathogen *A. solani*. The experiment was further conducted in *N. benthamiana* leaves with a similar result of ROS induction at 3 dpi (Figure 3C). Semi-quantitative RT-PCR confirmed the expression of *StTNLC7G2* in ROS-induced leaves (Figure 3D).

## Discussion

TNL genes are a subset of the NBS-LRR gene family. In the current work, a comprehensive analysis to identify the TNL members in potatoes utilizing *S. tuberosum* phureja genome resulted in 44 unique genes encoding 60 TNLs and 96 transcripts overall (including pseudogenes). The majority of genes was encoding more than one transcript. TNL gene frequency varies greatly across plant species, ranging from 0.27% in apples (Arya et al., 2014) to 0.000667% in common beans (Wu et al., 2017), while here we obtained a frequency of 0.117% in potatoes. Synteny and duplication assessment showed that all the genes are present in a single copy number except one (involved in tandem duplication). Probably higher fitness cost and more available transcripts per gene have reduced the duplication event occurrence. The analysis also identified the presence of the LRR domain in StTNLC7G2, based on CDD search that was omitted in the whole-genome analysis previously reported (Lozano et al., 2012). The physical distribution of *StTNL* genes identified two clusters at chr1 and chr11. As TNLs are known to form heterodimers to regulate the plant defense responses (Ashikawa et al., 2008), clustering of genes may help them to modulate simultaneously and co-expressed, if required. It has been reported that R-gene evolution is governed by cluster size, complexity, and duplication events (Friedman and Baker, 2007).

On average, TNL peptides were  ^~^ 500 amino acids in length with a coding sequence   ^~^ 3 kb. Exon–intron structures of these TNL varied significantly, and all of them were inclusive of introns (Supplementary Figure S3). Exons–introns exhibit a variety of structures that result in gene structural divergence due to three mechanisms: exon and intron distribution, insertion/deletion, and exonization/pseudo-exonization resulting in their gain or loss (Roy and Gilbert, 2005). In the present study, many transcripts were identified originating from a putative single gene, probably as a result of alternative splicing events due to the availability of multiple introns–exons. In one case, *StTNLC1G8* is encoding three transcripts (*StTNLG8T1*–*StTNLG8T3*). Such alternative splicing events have been reported in *Arabidopsis RPS4* (Borhan et al., 2004) and tobacco N (Whitham et al., 1994) due to the higher number of exons in their gene structure.

Many TNL transcripts showed higher expression in the presence of *A. solani*, including *StTNLC11G2*, which was also expressed in the early stages of resistant potato cultivar Beate against *Streptomyces turgidiscabies* (Dees et al., 2016). Another gene *StTNLC7G2* has been shown to express more in the resistant ‘Premier Russet’ variety of potatoes against PVY strain-O as compared with the susceptible ‘Russet Burbank’ variety (Goyer et al., 2015). The homolog of *StTNLC7G1* has been shown to upregulate in *Solanum cardiophyllum* and *Solanum pinnatisectum* against late blight attack in another case study (Gu et al., 2020).

The activation of H_2_O_2_-dependent responses in Solanaceae plants has been shown to successfully prevent the early growth of necrotrophic pathogens such as *Botrytis cinerea* (Asselbergh et al., 2007). Here, agroinfiltration of *StTNLC7G2* showed similar induction of ROS in response to the fungal pathogen as visualized by DAB staining, indicative of HR. Henceforth, all the data suggest functional involvement of *StTNLC7G2* in ROS-mediated plant innate immunity.

## Conclusion

The potato genome revealed the presence of 44 TNL genes. Chromosome 1 and chromosome 11 carried one TNL cluster each. Many TNL genes including *StTNLC7G2* were found to be getting modulated under pathogen attack with much higher expression in a tolerant variety. StTNLC7G2 provides resistance to the pathogen by inducing ROS at the site of the attack. The mechanism showed *StTNLC7G2* having a role in potato innate immunity. This work is the first attempt to understand the structural and functional insight of TNLs along with their characterization in the presence of *A. solani*.

## Data Availability

The original contributions presented in the study are included in the article/Supplementary Material, further inquiries can be directed to the corresponding author.
