# Supplementary material for: Genome-Wide Analysis of TIR-NBS-LRR Gene Family in Potato Identified StTNLC7G2 Inducing Reactive Oxygen Species in Presence of Alternaria solani
Source: Front Genet. 2022 Jan 10;12:791055. doi: 10.3389/fgene.2021.791055 (PMC8784597; doi:10.3389/fgene.2021.791055)
Supplement: Supplementary file 1 [file DataSheet2.pdf]

A

>GGGCTCCAACTATGAGGGCGGGCTGGCACCTCCCGGGGTGGCCAGCCTTGCTGAATTATTCCACCCGT
GTGTTTTGCGTACTTCTTGTTTCCTTGGTGGGCTCGCCCACCACAAGGACCAACCCATAAACCTTTTTG
CAATGGCAATCAGCGTCAGTAACAATGTAATAATTTACAACTTTCAACAACGGATCTCTTGGTTCTGGC
ATCGATGAAGAACGCAGCGAAATGCGATAAGTAGTGTGAATTGCAGAATTCAGTGAATCATCGAATCTT
TGAACGCACATTGCGCCCTTTGGTATTCCAAAGGGCATGCCTGTTCGAGCGTCATTTGTACCCTCAAGC
TTTGCTTGGTGTTGGGCGTCTTTTTGTCTCCCCTTGCGGGAGACTCGCCTTAAAGTCATTGGCAGCCGG
CCTACTGGTTTCGGAGCGCAGCACAAGTCGCGCTCTCTTCCAGCCCCAAGGTCTAGCATCCACCAAGCC
TTTTTTTTCAACTTTTGACCTCGGATCAGGTAGGGATACCCGCTGAACTTAAGCATATCAATAGGGCGG
AGGGA//

B

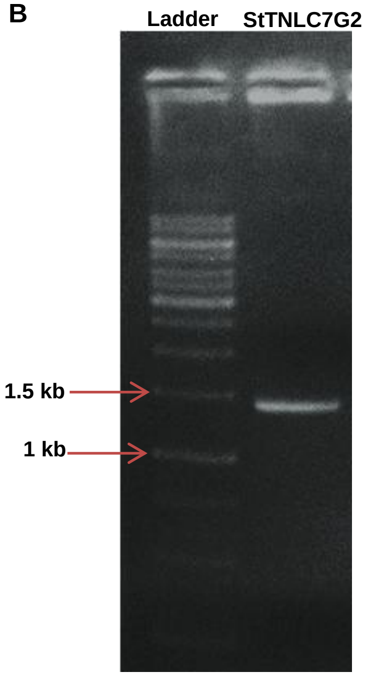

C

>MSSSSSFASNSQHYPRWK**YDVFLSFRGEDTRKTF**
MSHLYEGLKNRGIFTFQDDKRLEHGDSISEELLKA
IEDSQVAILIFSKNYATSRWCLNELVKIMECKKDE
NGKTVIPIFCDVDPSDVRNQRKIFAEAFAKHESNY
KDDVEGMQMVNGWKIALTAAANLKGYDVCDGIESE
NIQKIVDHISSRLCKSAYSLSSLQDVVG**IDAHLEK**
LKSQLQIEINDVRIVGIWGIGGVDKTTIAKSILDT
LSYQFEAACFLADVKENAKNNQLHTLQNALLSELL
RKKDDYVNNKYDGKCMIPSRLCSKKVLIVLDDIDH
SDHLEYLAGNLGWFGNGSRVIVTTRNRHLIEKDDA
IYEVPTLPDHEAMLLFNQHAFKKEVPDEHFKKFSL
EVENHAKGLPLALKVWGS**LLHKKGLTQWRRTVNLV**
KLCLVNQIKKNL//

sFig. 2. A) Sequencing result of ITS region of *Alternaria solani* B) Gel image showing amplification of 1296bp full length *StTNLC7G2* cds. C) Amino acid composition of domain TIR (Blue), NBS (Red) and LRR (Orange) in StTNLC7G2.
