# Supplementary material for: Genome-Wide Analysis of TIR-NBS-LRR Gene Family in Potato Identified StTNLC7G2 Inducing Reactive Oxygen Species in Presence of Alternaria solani
Source: Front Genet. 2022 Jan 10;12:791055. doi: 10.3389/fgene.2021.791055 (PMC8784597; doi:10.3389/fgene.2021.791055)
Supplement: Supplementary file 2 [file DataSheet4.pdf]

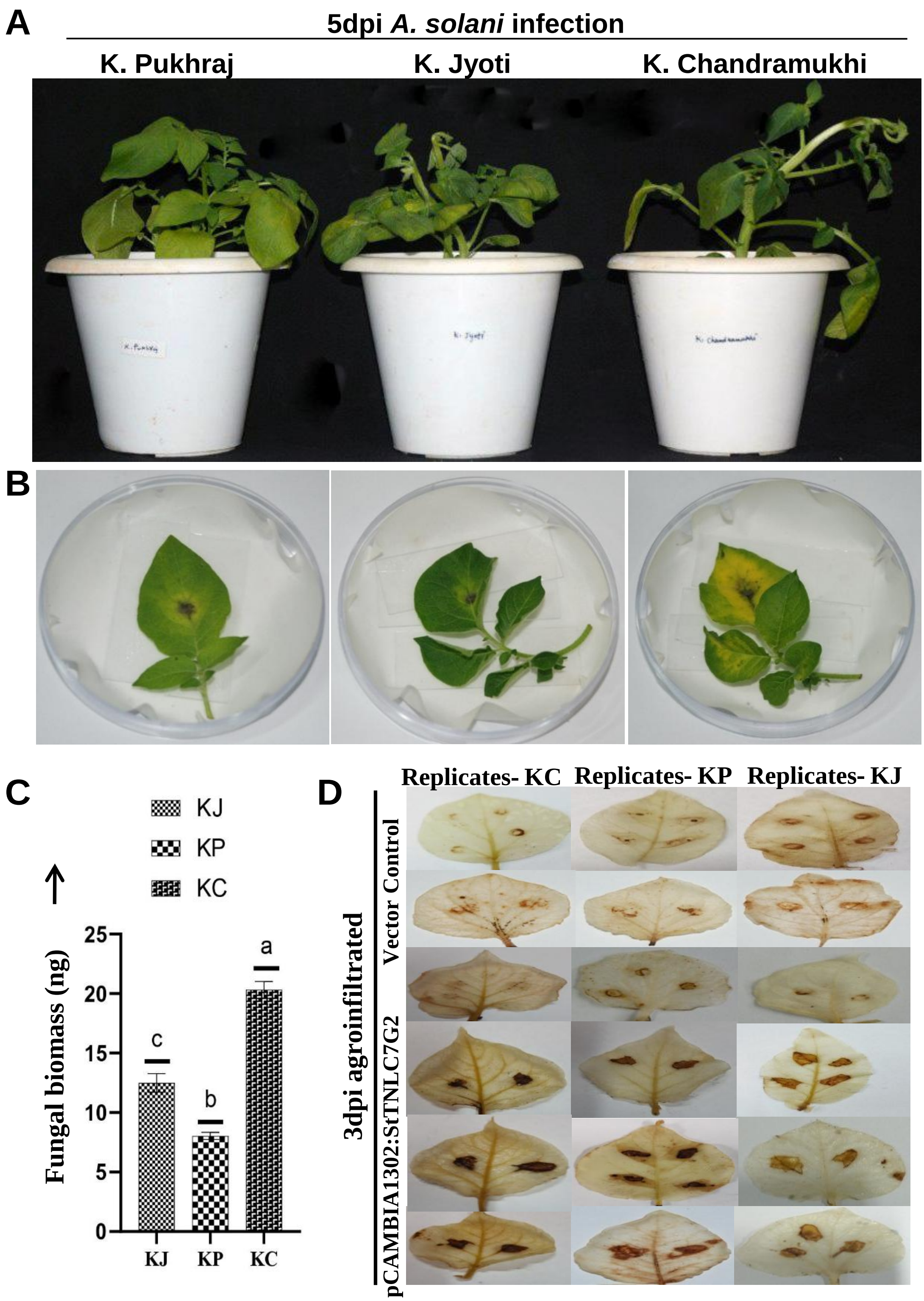

sFig.4 : Disease severity on selected potato varieties at 5 dpi A) On whole plants B) On detached leaf assay C) Fungal biomass measured using qRT-PCR D) Functional characterization of *StTNLC7G2* via agroinfiltration

Note: a,b,c represent significant differences in fungal biomass as tested using one-way ANOVA followed by a Duncan post hoc test for means ($p < 0.05$)
