# Supplementary material for: Genome-Wide Analysis of TIR-NBS-LRR Gene Family in Potato Identified StTNLC7G2 Inducing Reactive Oxygen Species in Presence of Alternaria solani
Source: Front Genet. 2022 Jan 10;12:791055. doi: 10.3389/fgene.2021.791055 (PMC8784597; doi:10.3389/fgene.2021.791055)
Supplement: Supplementary file 4 [file DataSheet1.pdf]

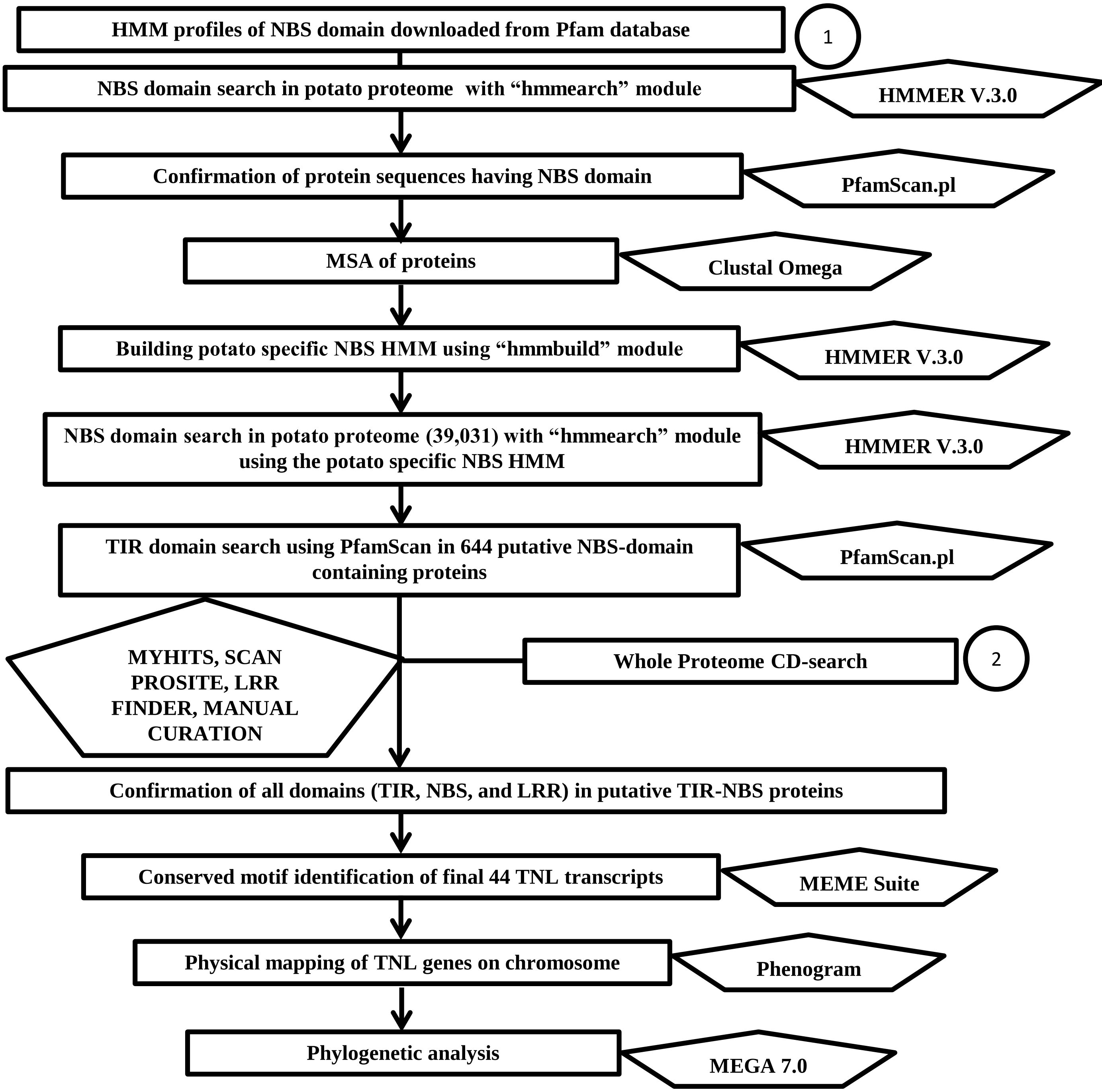

sFig. 1 : Pipeline illustrating the methodology used to identify the TNL domain containing transcripts
