# Supplementary material for: Genome-Wide Analysis of TIR-NBS-LRR Gene Family in Potato Identified StTNLC7G2 Inducing Reactive Oxygen Species in Presence of Alternaria solani
Source: Front Genet. 2022 Jan 10;12:791055. doi: 10.3389/fgene.2021.791055 (PMC8784597; doi:10.3389/fgene.2021.791055)
Supplement: Supplementary file 5 [file DataSheet5.pdf]

Table S1: Primer sequences used in the study

Primer sequences for the 14 transcripts and reference gene used in qRT-PCR			
**Transcript identity**	**Primer nomenclature**	**Primer sequence**	**Amplicon length (bp)**
StTNLC1G3T1	St_TNL5063_RT_1F	5′-AAACCAGGCTGTGAAGATG-3′	114
	St_TNL5063_RT_1R	5′-AGTAGGAGGAAGACGGACAG-3′	
StTNLCnG2T1	St_TNL6143_RT_1F	5′- ATCTCCCAACTGCCTCTCTC -3′	122
	St_TNL6143_RT_1R	5′- AGCCGTGTCAAGGAGGATAA -3′	
StTNLC4G3T1	St_TNL18619_RT_1F	5′-TCTGGCTGCTTGAAACTCG-3′	131
	St_TNL18619_RT_1R	5′-ATTGAAGGCCAGTGAGACG-3′	
StTNLC5G2T1	St_TNL32104_RT_1F	5′-CTTCTTCCATCGTCCGCTTG-3′	115
	St_TNL32104_RT_1R	5′-TCCAATGAGAGTAACCCGCC-3′	
StTNLC6G2T1	St_TNL4310_RT_1F	5′-GTAAGGAGGAAGGGAAGCGT-3′	131
	St_TNL4310_RT_1R	5′-GCCACTGCCAAACCAACTAA-3′	
StTNLC6G4T1	St_TNL4305_RT_1F	5′-CGAGGTGGTGCTTGAATGA-3′	157
	St_TNL4305_RT_1R	5′-ACGTCGATTCATGTTTGGC-3′	
StTNLC7G1T1	St_TNL53466_RT_1F	5′-ACCTCCGTCTTCCATTGTCC-3′	142
	St_TNL53466_RT_1R	5′-ATCGAGACGCAGAACATCCA-3′	
StTNLC7G2T1	St_TNL41857_RT_1F	5′- GCATGATTCCGAGCAGACTT-3′	189
	St_TNL41857_RT_1R	5′- GCCATTACCAAACCAGCCAA-3′	
StTNLC9G1T1	St_TNL45901_RT_1F	5′-TACAACCCGAGACGAGCATT-3′	87
	St_TNL45901_RT_1R	5′-TGTTGTGCTTCGTTGCCATT-3′	
StTNLC9G3T1	St_TNL13664_RT_1F	5′-CTTCGCTGTTACTGTTGCCC-3′	160
	St_TNL13664_RT_1R	5′-TGTTATCTGTTCCCCGCCTT-3′	
StTNLC11G7T1	St_TNL26554_RT_1F	5′-ATTGTTCGCCAGGAGTCAGT-3′	111
	St_TNL26554_RT_1R	5′-GCCTTCGATTGTTCCTGTACC-3′	
StTNLC11G8T1	St_TNL48338_RT_1F	5′-GGGACGAACACTTGCTGAAA-3′	121
	St_TNL48338_RT_1R	5′-TCTGGAGGGAGGGGATTCTT-3′	
StTNLC11G9T1	St_TNL1685_RT_1F	5′-AGCTGCCAGAATTTCCACA-3′	130
	St_TNL1685_RT_1R	5′-GTGGCAAGGAATCTGAAGC-3′	
StTNLC12G1T1	St_TNL51226_RT_1F	5′-GCTTATTCTTCGTGGCTGC-3′	82
	St_TNL51226_RT_1R	5′-TCTCCGAGGCTCTTCAAGT-3′	
Elongation factor 1α	ef1α_RT_1F	5′-GATGGTCAGACCCGTGAACA-3′	106
	ef1α_RT_1R	5′-CCTTGGAGTACTTCGGGGTG-3′	
Nb_Elongation factor 1α	ef1α_RT_1F	5′- CCCCTACGTCTTCCACTTCA-3′	117
	ef1α_RT_1R	5′- ATACCAGGCTTGAGGACACC-3′	
**Primer set for full length gene amplification selected for agroinfiltration**			
StTNLC7G2	St_TNLpcam41857NCOI_1Fb	5′-CATGCCATGGTCATCATCTTCCTCTTTTGCG-3′	
	St_TNLpcam41857BstEII_1Rb	5′-CCGGTCACCCTACAAGTTTTTCTTTATTTGGTT-3′	

	St_TNLpcam41857BGIII _1Fb	5′-GGAAGATCTCAAGTTTTTCTTTATTTGGTTTAC-3′	
ITS	ITS-1	5′-TCCGTAGGTGAACCTGCGG-3′	
	ITS-4	5′-TCCTCCGCTTATTGATATGC-3′	
AS1	AS1_F	5′-GCTCCCACTCCTTCCGCGC-3′	289
AS2	AS2_R	5′-GGAGGTGGAGTTACCGACAA-3′	

Table S2: All the *TNLs* that identified in the study encoding three domains TIR, NBS and LRR as predicted from available *S. tuberosum* genome.

S. No.	Chromosome No.	Gene id	Protein id	Domain present	Nomenclature
1.	1	PGSC0003DMG400022699	PGSC0003DMP400039354	TNL	StTNLC1G1P1
2.		PGSC0003DMG400024055	PGSC0003DMP400041617	TNL	StTNLC1G2P1
			PGSC0003DMP400041616	TNL	StTNLC1G2P2
3.		PGSC0003DMG400002799	PGSC0003DMP400005063	TNL	StTNLC1G3P1
4.		PGSC0003DMG402018257	PGSC0003DMP400031808	TNL	StTNLC1G4P1
			PGSC0003DMP400031809	TNL	StTNLC1G4P2
5.		PGSC0003DMG400018256	PGSC0003DMP400031803	TNL	StTNLC1G5P1
			PGSC0003DMP400031802	TNL	StTNLC1G5P2
6.		PGSC0003DMG400018216	PGSC0003DMP400031726	TNL	StTNLC1G6P1
7.		PGSC0003DMG400013098	PGSC0003DMP400023191	TNL	StTNLC1G7P1
			PGSC0003DMP400023192	TNL	StTNLC1G7P2
8.		PGSC0003DMG400020722	PGSC0003DMP400036044	TNL	StTNLC1G8P1
			PGSC0003DMP400036043	TNL	StTNLC1G8P2
			PGSC0003DMP400036045	TNL	StTNLC1G8P3
9.		PGSC0003DMG401020721	PGSC0003DMP400036041	TNL	StTNLC1G9P1
10.	2	PGSC0003DMG400013090	PGSC0003DMP400023175	TNL	StTNLC2G1P1
11.	4	PGSC0003DMG400006003	PGSC0003DMP400010670	TNL	StTNLC4G1P1
			PGSC0003DMP400010669	TNL	StTNLC4G1P2
12.		PGSC0003DMG400020935	PGSC0003DMP400036348	TNL	StTNLC4G2P1
13.		PGSC0003DMG400010527	PGSC0003DMP400018619	TNL	StTNLC4G3P1
14.	5	PGSC0003DMG400018428	PGSC0003DMP400032105	TNL	StTNLC5G1P1
15.		PGSC0003DMG400018429	PGSC0003DMP400032104	TNL	StTNLC5G2P1
16.		PGSC0003DMG401022603	PGSC0003DMP400039170	TNL	StTNLC5G3P1
17.		PGSC0003DMG400002357	PGSC0003DMP400004210	TNL	StTNLC5G4P1
18.	6	PGSC0003DMG400009686	PGSC0003DMP400017113	TNL	StTNLC6G1P1
19.		PGSC0003DMG402002428	PGSC0003DMP400004310	TNL	StTNLC6G2P1
20.		PGSC0003DMG400002427	PGSC0003DMP400004308	TNL	StTNLC6G3P1
			PGSC0003DMP400004307	TNL	StTNLC6G3P2
21.		PGSC0003DMG400002426	PGSC0003DMP400004305	TNL	StTNLC6G4P1
22.		PGSC0003DMG400026104	PGSC0003DMP400045297	TNL	StTNLC6G5P1
23.		PGSC0003DMG400031318	PGSC0003DMP400054560	TNL	StTNLC6G6P1
24.	7	PGSC0003DMG401030700	PGSC0003DMP400053466	TNL	StTNLC7G1P1
25.		PGSC0003DMG400024206	PGSC0003DMP400041857	TNL	StTNLC7G2P1
			PGSC0003DMP400041858	TNL	StTNLC7G2P2
26.		PGSC0003DMG400013543	PGSC0003DMP400023970	TNL	StTNLC7G3P1
			PGSC0003DMP400023971	TNL	StTNLC7G3P2

			PGSC0003DMP400023972	TNL	StTNLC7G3P3
27.		PGSC0003DMG400017317	PGSC0003DMP400030257	TNL	StTNLC7G4P1
28.	8	PGSC0003DMG400018461	PGSC0003DMP400032156	TNL	StTNLC8G1P1
29.	9	PGSC0003DMG401026432	PGSC0003DMP400045901	TNL	StTNLC9G1P1
30.		PGSC0003DMG400026433	PGSC0003DMP400045909	TNL	StTNLC9G2P1
			PGSC0003DMP400045910	TNL	StTNLC9G2P2
			PGSC0003DMP400045911	TNL	StTNLC9G2P3
31.		PGSC0003DMG400007743	PGSC0003DMP400013664	TNLNL	StTNLC9G3P1
32.	11	PGSC0003DMG402016979	PGSC0003DMP400029664	TNL	StTNLC11G1P1
33.		PGSC0003DMG402016981	PGSC0003DMP400029677	TNL	StTNLC11G2P1
34.		PGSC0003DMG403016981	PGSC0003DMP400029678	TNL	StTNLC11G3P1
35.		PGSC0003DMG400015693	PGSC0003DMP400027528	TNL	StTNLC11G4P1
36.		PGSC0003DMG401015682	PGSC0003DMP400027502	TNL	StTNLC11G5P1
			PGSC0003DMP400027485	TNL	StTNLC11G5P2
37.		PGSC0003DMG400015681	PGSC0003DMP400027484	TNL	StTNLC11G6P1
38.		PGSC0003DMG400015128	PGSC0003DMP400026554	TNL	StTNLC11G7P1
39.		PGSC0003DMG400027797	PGSC0003DMP400048338	TNL	StTNLC11G8P1
40.		PGSC0003DMG400000888	PGSC0003DMP400001685	LNTNXL	StTNLC11G9P1
41.		PGSC0003DMG400033334	PGSC0003DMP400055979	TNL	StTNLC11G10P1
			PGSC0003DMP400055980	TNL	StTNLC11G10P2
42.	12	PGSC0003DMG400029415	PGSC0003DMP400051226	TNL	StTNLC12G1P1
43.	Unanchored	PGSC0003DMG400021887	PGSC0003DMP400037911	TNL	StTNLCnG1P1
			PGSC0003DMP400037912	TNL	StTNLCnG1P2
44.	Unanchored	PGSC0003DMG400003445	PGSC0003DMP400006143	TNL	StTNLCnG2P1

*No TNL sequence observed in Chr3 and Chr10.

Table S3: TNL genes that encode truncated peptides other than full length TNL proteins

S. No.	Chromosome No.	Gene id	Protein id	Domain present
1.	1	PGSC0003DMG400002799	PGSC0003DMP400005061	N
			PGSC0003DMP400005062	N
2.		PGSC0003DMG402018257	PGSC0003DMP400031806	T
			PGSC0003DMP400031807	N
			PGSC0003DMP400031810	N
3.		PGSC0003DMG400018216	PGSC0003DMP400031727	No CD
4.		PGSC0003DMG400013098	PGSC0003DMP400023190	L
5.		PGSC0003DMG400020722	PGSC0003DMP400036042	No CD
6.		PGSC0003DMG401020721	PGSC0003DMP400036038	T
7.	4	PGSC0003DMG400010527	PGSC0003DMP400018620	No CD
8.	5	PGSC0003DMG401022603	PGSC0003DMP400039169	T
9.	6	PGSC0003DMG400002426	PGSC0003DMP400004304	T
10.		PGSC0003DMG400026104	PGSC0003DMP400045298	No CD
11.	7	PGSC0003DMG401030700	PGSC0003DMP400053468	NL
			PGSC0003DMP400053469	N
			PGSC0003DMP400053470	DUF
12.		PGSC0003DMG400024206	PGSC0003DMP400041852	L
			PGSC0003DMP400041853	L
			PGSC0003DMP400041854	L
			PGSC0003DMP400041855	L
			PGSC0003DMP400041856	L
			PGSC0003DMP400041859	L
13.		PGSC0003DMG400013543	PGSC0003DMP40002369	T
14.	9	PGSC0003DMG401026432	PGSC0003DMP400045905	L
			PGSC0003DMP400045906	L
			PGSC0003DMP400045907	No CD
15.	11	PGSC0003DMG402016979	PGSC0003DMP400029665	L
			PGSC0003DMP400029666	L
16.		PGSC0003DMG403016981	PGSC0003DMP400029681	L
17.		PGSC0003DMG400015681	PGSC0003DMP400027483	L
18.		PGSC0003DMG400015128	PGSC0003DMP400026555	N
			PGSC0003DMP400026556	No CD
19.		PGSC0003DMG400033334	PGSC0003DMP400055976	L
			PGSC0003DMP400055977	L
			PGSC0003DMP400055978	NL
20	12	PGSC0003DMG400029415	PGSC0003DMP400051225	TL

*N: NBS; T: TIR; TL: TIR-NBS; NL: NBS-LRR; L: LRRs

Table S4: Constitutive motifs present within the domains of TNL proteins

S.No.	Motif Sequence	Signature motif logo	Width	Occurrence	Description
1.	YDVFLSFRGEDTRKTFTSHLYEGLKNRGI	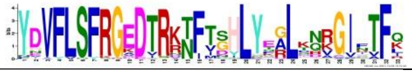	29	84%	TIR-1
2.	DDKRLERGDSISEELLKAIEESQVAJIVFSKNYATSRWCLBELVKIMECK	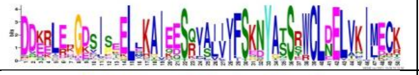	50	84%	TIR-2
3.	TVJPVFYDVDPSHVRKQSESFAEAFAKHE	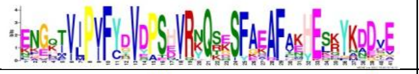	29	84%	TIR-3
4.	KVQGWRTALTAAABLKGYDJRBGIES	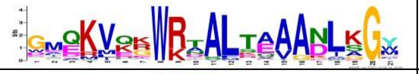	26	84%	TIR-4
5.	VRIVGIWGMGGVGKTTJAKAIFBTJSSQF	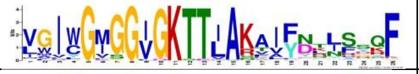	29	84%	P-Loop
6.	HSLQNILLSELLREKDBYVNN	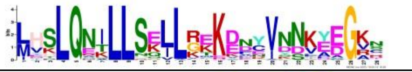	21	82%	RNBS-A
7.	RLCSKKVLIVLDDVD	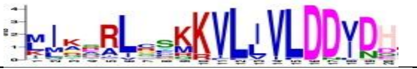	15	82%	KIN-2
8.	QLEYLAGDRDWFGNGSRIIITTRB	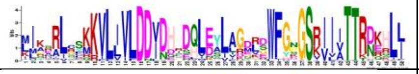	24	82%	RNBS-B
9.	LFSQHAFKKEVPDE	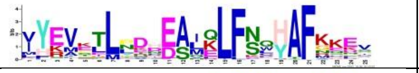	14	84%	RNBS-C
10.	EVVSHAKGLPLALKVLGSSL	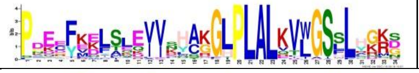	20	84%	GLPL
11.	LEDEEQEIFLDIACFFRGKEKDEVMQILESCDFGAEI	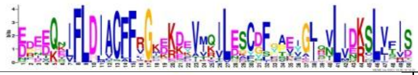	37	82%	RNBS-D
12.	YBKIZMHDLIQDMGRYIVRMZKDSGPRSR	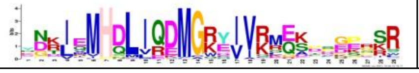	29	84%	MHDL

Table S5: Duplication events detail in StTNLs

Gene Id	Position	Gene Id	Position
**Segmental duplication TNL to all**			
PGSC0003DMG400002217	Chr 4	PGSC0003DMG402002428	Chr 6
PGSC0003DMG400002426	Chr 6	PGSC0003DMG400004146	Chr 4
PGSC0003DMG400002426	Chr 6	PGSC0003DMG400016654	Chr 6
PGSC0003DMG400002426	Chr 6	PGSC0003DMG400026792	Chr 9
PGSC0003DMG400002426	Chr 6	PGSC0003DMG400033667	Chr 6
PGSC0003DMG402002428	Chr 6	PGSC0003DMG400003356	Chr 5
PGSC0003DMG402002428	Chr 6	PGSC0003DMG400019418	Chr 10
PGSC0003DMG402002428	Chr 6	PGSC0003DMG400019928	Chr 1
PGSC0003DMG402002428	Chr 6	PGSC0003DMG400024231	Chr 4
PGSC0003DMG402002428	Chr 6	PGSC0003DMG400039567	Chr 0
PGSC0003DMG401003025	Chr 5	PGSC0003DMG400021887	Chr 0
PGSC0003DMG400003356	Chr 5	PGSC0003DMG400020935	Chr 4
PGSC0003DMG400003356	Chr 5	PGSC0003DMG400021887	Chr 0
PGSC0003DMG400003445	Chr 0	PGSC0003DMG402011117	Chr 10
PGSC0003DMG400003445	Chr 0	PGSC0003DMG400023198	Chr 0
PGSC0003DMG400003445	Chr 0	PGSC0003DMG400030267	Chr 0
PGSC0003DMG400007743	Chr 9	PGSC0003DMG400011109	Chr 10
PGSC0003DMG400007743	Chr 9	PGSC0003DMG400016601	Chr 9
PGSC0003DMG400007743	Chr 9	PGSC0003DMG402020721	Chr 1
PGSC0003DMG400008394	Chr 10	PGSC0003DMG400020935	Chr 4
PGSC0003DMG400010527	Chr 4	PGSC0003DMG400030267	Chr 0
PGSC0003DMG400010527	Chr 4	PGSC0003DMG400034527	Chr 0
PGSC0003DMG400010527	Chr 4	PGSC0003DMG400043376	Chr 4
PGSC0003DMG400015681	Chr 11	PGSC0003DMG400020728	Chr 1
PGSC0003DMG401015682	Chr 11	PGSC0003DMG400018213	Chr 1
PGSC0003DMG400015693	Chr 11	PGSC0003DMG402018257	Chr 1
PGSC0003DMG401016933	Chr 4	PGSC0003DMG400021887	Chr 0
PGSC0003DMG400017317	Chr 7	PGSC0003DMG400023645	Chr 4
PGSC0003DMG400018213	Chr 1	PGSC0003DMG400026104	Chr 6
PGSC0003DMG402018257	Chr 1	PGSC0003DMG401022603	Chr 5
PGSC0003DMG400019928	Chr 1	PGSC0003DMG400021887	Chr 0
PGSC0003DMG400020728	Chr 1	PGSC0003DMG401026432	Chr 9
PGSC0003DMG400020935	Chr 4	PGSC0003DMG400024206	Chr 7
PGSC0003DMG400021887	Chr 0	PGSC0003DMG400024055	Chr 1
PGSC0003DMG400021887	Chr 0	PGSC0003DMG400034527	Chr 0
**Segmental duplication TNL to TNL**			
PGSC0003DMG402002428	Chr 6	PGSC0003DMG400021887	Chr 0
PGSC0003DMG400003445	Chr 0	PGSC0003DMG400022699	Chr 1
PGSC0003DMG400009686	Chr 6	PGSC0003DMG400020935	Chr 4
PGSC0003DMG400010527	Chr 4	PGSC0003DMG400020935	Chr 4
PGSC0003DMG400013090	Chr 2	PGSC0003DMG400020935	Chr 4
PGSC0003DMG400017317	Chr 7	PGSC0003DMG400022699	Chr 1
PGSC0003DMG400020935	Chr 4	PGSC0003DMG400021887	Chr 0
**Tandem duplication TNL to TNL**			
PGSC0003DMG401026432	Chr 9	PGSC0003DMG400026433	Chr9

Table S6: Expression values of StTNL transcripts at different interval in Kufri Chandramukhi post infection

Transcript nomenclature	Kufri Chandramukhi				
	**1_DPI**	**2_DPI**	**3_DPI**	**5_DPI**	**7_DPI**
**StTNLC1G3T1**	5.334764224±0.129396803	5.293139924±0.387982692	5.009590369±0.436675082	5.324107362±0.434841759	5.260055339±0.054687917
**StTNLCnG2T1**	7.379192564±0.510669072	5.655202129±0.449834707	5.660625241±0.83739393	5.369074396±0.148353728	5.021525008±0.330185211
**StTNLC4G3T1**	6.762246219±0.397954349	5.832803545±0.443869238	5.258319131±0.301634578	5.062377409±0.23980831	3.333418568±0.521701645
**StTNLC5G2T1**	5.048480346±0.453686926	4.144641464±0.172271482	3.955102529±0.342087536	4.132455717±0.063040726	4.100221072±0.12785109
**StTNLC6G2T1**	7.250726565±0.476764066	4.612102292± 0.122558121	2.705440763± 0.634910915	2.671880719± 0.149130037	2.865633595± 0.31651234
**StTNLC6G4T1**	4.713989676±0.244841124	4.657371628±0.392610919	4.71039872±0.42330487	4.184112242±0.048864902	4.126955773±0.04290727
**StTNLC7G1T1**	5.661981782±0.528278192	5.530537017±0.561747687	5.290268848±0.012237204	5.217877101±0.361097564	4.25962801±0.227801245
**StTNLC7G2T1**	6.255955393±0.904245174	3.428321829±0.193170734	3.16559196±0.276338882	2.308466427±0.579391964	2.178048406±0.952722613
**StTNLC9G1T1**	6.997221725±0.67689123	6.707844219±0.602807383	6.019688239±0.585833355	4.610697179±0.382625017	4.345669898±0.345669898
**StTNLC9G3T1**	5.902422146±0.550711927	5.815861381±0.68215779	5.187303981±0.430475521	4.037809892±0.170278512	2.343748237±0.240810181
**StTNLC11G7T1**	5.187786444±0.959714281	4.343051651± 0.161678207	2.178693066± 0.290502128	1.770131748± 0.284881275	0.618116352± 0.283501653
**StTNLC11G8T1**	6.454529317±0.134198822	7.341530965± 0.316566059	5.89535459± 0.382885686	3.285773362± 0.873022848	2.985816634± 0.196329301
**StTNLC11G9T1**	7.443845599±0.283645032	6.605477899±0.099369012	6.521557694±0.112998673	6.218056727±0.279962444	6.038496918±0.459522253
**StTNLC12G1T1**	6.797520817±0.023558318	5.234814912±0.703279371	4.982772131±0.258801485	4.623613738±0.166735761	4.130921882±0.185991064

.

Table S7: Expression values of StTNL transcripts at different interval in Kufri Jyoti post infection.

Transcript nomenclature	Kufri Jyoti				
	1_DPI	2_DPI	3_DPI	5_DPI	7_DPI
**StTNLC1G3T1**	5.426939±0.075229	5.446497±0.204923	6.350845±0.329858	7.235481±0.540951	7.664149±0.503948
**StTNLCnG2T1**	5.375742±0.242038	5.755674±0.477642	7.260153±0.049976	9.211672±0.637484	9.29761±0.108023
**StTNLC4G3T1**	6.707369±0.431278	7.184451±0.73725	7.569188±0.460373	9.650325±0.193069	12.92612±0.716042
**StTNLC5G2T1**	5.877557±0.10184	5.910624±0.632592	7.212132±0.362159	7.621287±0.052826	8.130311±0.870157
**StTNLC6G2T1**	7.580403±0.516155	10.79444±0.636678	12.21741±0.423254	13.92376±0.621038	16.17509±0.504364
**StTNLC6G4T1**	7.253502±0.272976	7.729679±0.668055	10.06795±0.488117	13.13573±0.318612	14.5715±0.151498
**StTNLC7G1T1**	6.195871±0.47201	6.137834±0.519966	8.75456±0.060681	6.088337±0.895901	6.161623±0.659456
**StTNLC7G2T1**	6.264018±0.660176	6.998029±0.363472	10.60841±0.191081	11.47192±0.079516	13.99787±0.252132
**StTNLC9G1T1**	7.235426±0.075226	8.86503±0.582911	9.729005±0.610355	10.36379±0.104549	11.43202±0.03962
**StTNLC9G3T1**	5.93636±0.472199	8.892295±0.369596	8.908877±0.325524	9.323465±0.322997	10.19746±0.141358
**StTNLC11G7T1**	6.200805±0.386842	8.742388±0.90738	9.800160842±0.610574	10.83618±0.863143	11.24231±0.389473
**StTNLC11G8T1**	6.502351±0.961913	8.481691±0.224101	8.668015±0.270282	9.735234±0.639882	10.16176±0.086263
**StTNLC11G9T1**	7.86639±0.245286	8.111871±0.056226	9.669035±0.916059	13.43107±0.818564	17.97362±0.506789
**StTNLC12G1T1**	4.244815±0.13236	4.548699±0.173347	5.033973±0.244058	5.543766±0.192055	5.979541±0.11609

Table S8: Expression values of StTNL transcripts at different interval in Kufri Pukhraj post infection.

**Transcript nomenclature**	**Kufri Pukhraj**				
	**1_DPI**	**2_DPI**	**3_DPI**	**5_DPI**	**7_DPI**
**StTNLC1G3T1**	6.129294±0.552915	6.330303±0.410125	7.542322±0.02614	9.497202±0.558905	11.28894±0.546742
**StTNLCnG2T1**	6.212684±0.107647	7.621836±0.105654	8.136537±0.926534	8.635693±0.179548	8.955695±0.557962
**StTNLC4G3T1**	5.386001±0.419406	7.25772±0.484354	10.56519±0.358557	10.79444±0.597958	12.73147±0.863997
**StTNLC5G2T1**	5.389452±0.074708	7.594783±0.026321	9.12611±0.160916	11.71269±0.674787	11.85914±0.677561
**StTNLC6G2T1**	7.71563±0.799332	8.23755924±0.2863	12.648251±0.438179	12.88394±0.479758	15.09189±0.470587
**StTNLC6G4T1**	5.955904±0.32242	7.562452±0.835281	11.13437±0.578311	12.31893±0.7675	12.91402±0.36529
**StTNLC7G1T1**	4.907489±0.407255	5.761577±0.25941	5.978529±0.275866	5.755769±0.019948	5.740782±0.238615
**StTNLC7G2T1**	6.954998±0.213718	8.12415±0.450037	8.605247±0.149103	10.29989±0.759768	11.43257±0.118863
**StTNLC9G1T1**	5.132659±0.956849	6.32212±0.546403	7.260153±0.050674	8.833164±0.546381	9.101145±0.346796
**StTNLC9G3T1**	8.650631±0.797295	9.051303±0.595159	9.63856±0.700263	10.30609±0.295854	12.0856±0.209406
**StTNLC11G7T1**	6.631931±0.285876	7.495622±0.285618	8.204775±0.369413	8.220365±0.880279	9.927369±0.726418
**StTNLC11G8T1**	5.006760±0.500313	5.586971±0.765788	6.499859±0.561765	6.894398±0.167226	7.111425±0.098579
**StTNLC11G9T1**	7.018681±0.87118	7.108412±0.590469	8.000192±0.424673	9.828346±0.510477	10.24087±0.682755
**StTNLC12G1T1**	6.260746±0.368278	7.379193±0.510669	8.759608±0.292847	9.828982±0.193096	10.12703±0.140381
